# Heterogeneous Enhancement Pattern in DCE-MRI Reveals the Morphology of Normal Lymph Nodes: An Experimental Study

**DOI:** 10.1155/2019/4096706

**Published:** 2019-04-04

**Authors:** Pietro Bontempi, Alice Busato, Giamaica Conti, Sabino Walter Della Sala, Pasquina Marzola, Paolo Farace

**Affiliations:** ^1^Proton Therapy Department, S. Chiara Hospital, Trento, Italy; ^2^Department of Computer Science, University of Verona, Verona, Italy; ^3^Department of Neuroscience and Biomedicine, University of Verona, Verona, Italy; ^4^Radiology Department, S. Maria del Carmine Hospital, Rovereto, Italy

## Abstract

**Purpose:**

To investigate the heterogeneous enhancement pattern in normal lymph nodes of healthy mice by different albumin-binding contrast agents.

**Methods:**

The enhancement of normal lymph nodes was assessed in mice by dynamic contrast-enhanced MRI (DCE-MRI) after the administration of two contrast agents characterized by different albumin-binding properties: gadopentetate dimeglumine (Gd-DTPA) and gadobenate dimeglumine (Gd-BOPTA). To take into account potential heterogeneities of the contrast uptake in the lymph nodes, k-means cluster analysis was performed on DCE-MRI data. Cluster spatial distribution was visually assessed. Statistical comparison among clusters and contrast agents was performed on semiquantitative parameters (AUC, wash-in rate, and wash-out rate) and on the relative size of the segmented clusters.

**Results:**

Cluster analysis of DCE-MRI data revealed at least two main clusters, localized in the outer portion and in the inner portion of each lymph node. With both contrast agents, AUC (*p* < 0.01) and wash-in (*p* < 0.05) rates were greater in the inner cluster, which also showed a steeper wash-out rate than the outer cluster (Gd-BOPTA, *p* < 0.01; Gd-DTPA, *p*=0.056). The size of the outer cluster was greater than that of the inner cluster by Gd-DTPA (*p* < 0.05) and Gd-BOPTA (*p* < 0.01). The enhancement pattern of Gd-DTPA was not significantly different from the enhancement pattern of Gd-BOPTA.

**Conclusion:**

DCE-MRI in normal lymph nodes shows a characteristic heterogeneous pattern, discriminating the periphery and the central portion of the lymph nodes. Such a pattern deserves to be investigated as a diagnostic marker for lymph node staging.

## 1. Introduction

Dynamic contrast-enhanced MRI (DCE-MRI) has been widely used to quantify tissue perfusion in preclinical [1, 2] and clinical studies. In DCE-MRI, multiple T1-weighted images are acquired before and at different time points after the administration of a Gd-based contrast agent, allowing the quantification of parameters related to perfusion [1, 2]. The diagnostic usefulness of DCE-MRI in tumors largely relies on the peculiar features of the tumor tissue: increased blood volume fraction and vessel permeability.

Thanks to high-resolution T1-weighted images, DCE-MRI has been extensively applied in clinical studies to characterize the breast [3] and solitary pulmonary nodules [4] and in general in cancer diagnosis including the prostate [5] and liver [6]. Moreover, DCE-MRI can have a role for the detection and characterization of lymph nodes. Quantitative or semiquantitative parameters extracted from DCE-MRI data (describing, for example, the uptake and wash-out of the contrast agent) are expected to discriminate between positive and negative nodes. Accordingly, DCE-MRI showed promising performance to assess suspicious lymph nodes at different sites, such as the head and neck [7–9], rectal [10, 11], and cervical [12–14] and in the axillary lymph nodes in breast cancer patients [15–19], for which the role of MRI, including DCE-MRI, was recently reviewed [20].

The overarching aim of our study is to define the diagnostic potential of DCE-MRI for lymph node staging. For this purpose, the first step is investigation of normal lymph nodes since the definition of the enhancement pattern of normal nodal tissue is a needed preparatory knowledge to reliably detect alterations induced by metastasization. The aim of the present study is therefore obtaining the definition of the enhancement pattern in DCE-MRI of normal lymph nodes through systematic investigation in healthy mice. A further aim is to develop a semiautomatic and operator-independent image analysis procedure by using cluster analysis of DCE-MRI enhancement patterns.

Moreover, Gd-based contrast agents can have different binding properties to albumin and consequently different capability to reveal blood vessels [21, 22], potentially generating a different lymphatic uptake/drainage pattern. In order to investigate if and how different protein binding may affect DCE-MRI findings in normal lymph nodes, two Gd-based contrast agents were administered, namely (i) gadopentetate dimeglumine (Gd-DTPA) and (ii) gadobenate dimeglumine (Gd-BOPTA). Specifically, Gd-BOPTA can be classified as an albumin-bound contrast agent [23], while Gd-DTPA is a non-albumin-bound extracellular contrast agent.

## 2. Methods

### 2.1. MRI Acquisition

Athymic nude mice were anesthetized by inhalation of a mixture of N_2_ and O_2_ containing 0.5–1% isoflurane (Forane, Abbott) and were cannulated in the tail vein for contrast agent injection during DCE-MRI scan. A single birdcage coil (3.5 cm i.d.) configuration was used for radiofrequency excitation and MRI signal detection. Images were acquired using a BioSpec tomograph (Bruker, Karlsruhe, Germany) equipped with a 4.7 T, 33 cm bore horizontal magnet (Oxford Ltd., Oxford, United Kingdom). For the imaging session, mice were placed in a prone position over a heated bed; body temperature and respiratory rate were monitored by using an MRI-compatible physiological monitor (PC-SAM, Small Animal Instruments, Inc., NY).

Before the administration of the contrast agent, a standard non-fat-suppressed T1-weighted sequence (multislice RARE with TR/TE: 550/7.6 ms, flip angle: 180°, matrix size: 175 × 100, number of slices: 6, field-of-view: 3.5 × 2.0 mm^2^, slice thickness: 0.5 mm, number of averages: 8, and RARE factor: 8) was applied to investigate the presence of a fatty hilum, which has been often reported in human lymph nodes [24, 25].

Afterwards, a dynamic series of multislice T1-weighted RARE images were acquired with fat suppression and with the following parameters: TR/TE: 550/7.6 ms, flip angle: 180°, matrix size: 175 × 100, number of slices: 6, field-of-view: 3.5 × 2.0 mm^2^, slice thickness: 0.5 mm, number of averages: 8, RARE factor: 8, number of scans: 70, and total acquisition time: approximately 46 minutes. Two Gd-based contrast agents were tested in different sessions, by bolus injection (100 *µ*mol/kg) during the time interval between the third and fourth scan of the dynamic series: (i) Gd-DTPA and (ii) Gd-BOPTA.

DCE-MRI was performed in total on 9 healthy mice (5 mice were injected by Gd-DTPA and 4 by Gd-BOPTA).

The experimental plan received authorization from the Italian Ministry of Health (approval number: 676/2018-PR) and was approved by the Animal Care and Use Committee of the University of Verona. Animal work was conducted following the Italian law (D.L. no. 26 of 4 March 2014) and the European Union normative (2010/63/EU). Major efforts were performed to minimize the number of animals and to avoid their suffering.

### 2.2. Image Analysis

Some DCE-MRI scans were affected by peristalsis and breath-induced motion. To compensate for these displacements, a linear registration approach was adopted by means of the MCFLIRT tool of FSL [26]. Even though that tool is designed for brain imaging, it proved to be effective even in the abdominal region. The 2^nd^ volume after the contrast agent injection was used as a reference image for the registration of the whole time series. When the linear registration approach was not sufficient, a nonlinear approach was adopted, carrying out nonlinear registration by the FNIRT tool of FLS and finely tuning the registration parameters to obtain a “gentle” warp field.

The subsequent postprocessing was performed by MATLAB (MathWorks®, Natick, MA). For each node, normalized differential enhancement (NDE) curves were calculated on a pixel-by-pixel basis, subtracting the signal intensity before contrast injection (SI_PRE_) from the signal intensity at a given time point SI(*t*) and normalizing it by (DE_MAX_)_MUSCLE_, that is, the maximal differential enhancement over an area drawn on the tight muscle:

(1) $$\begin{array}{c} \text{NDE}\left(t\right)=\frac{\left[\text{SI}\left(t\right)-{\text{SI}}_{\text{PRE}}\right]}{{\left({\text{DE}}_{\text{MAX}}\right)}_{\text{MUSCLE}}}. \end{array}$$

NDE was normalized by the signal intensity of the muscle to compensate, at least partially, any possible difference in the effectively administered dosage of the contrast agents.

For each lymph node, the central slice was identified and a ROI was manually drawn to cover the entire nodal tissue.

To investigate the enhancement pattern in the nodes, cluster analysis was performed pixel by pixel on the NDE curves by means of a k-means algorithm. This algorithm requires as an input the number of clusters to be identified, and the minimum value (two clusters) was chosen to detect potential heterogeneities. To verify whether this arbitrary choice could be appropriate, principal component analysis (PCA) was performed on the same set of NDE curves, to obtain the data variance explanation as a function of the number of components.

Segmented colour-coded maps were obtained for each lymph node, to visualize the spatial distribution of each cluster obtained by cluster analysis.

After cluster analysis, in each lymph node, the NDE curves of each pixel belonging to a specific cluster were averaged to obtain the cluster-averaged NDE curves. Semiquantitative parameters were then extracted from these curves, namely, area under the curve (AUC), wash-in rate, and wash-out rate. The wash-in rate was defined as the maximum slope of the NDE curve between two consecutive time points comprised between the last baseline point and the point of maximal enhancement [27]. The wash-out rate was defined as the slope of the line that best fits the last 40 time points (i.e., scans 31 – 70) of the enhancement curve [21]. Both wash-out and wash-in rates were normalized by the maximal enhancement in each lymph node.

Statistical differences in the semiquantitative curve parameters (between clusters and between contrast agents) were assessed by two-way ANOVA, and the results were corrected for multiple comparisons with the Bonferroni method.

### 2.3. Histological Analysis

For histological investigation, lymph nodes were excised and fixed in 4% paraformaldehyde for four hours. After fixation and embedding in paraffin, 7 *μ*m thick sections were cut and stained with hematoxylin and eosin (H&E). The sections were observed using an optical microscope (Olympus BX63; Life Science Solutions, Centre Valley, PA) at 4x and 10x magnifications.

## 3. Results

**Figure 1:**
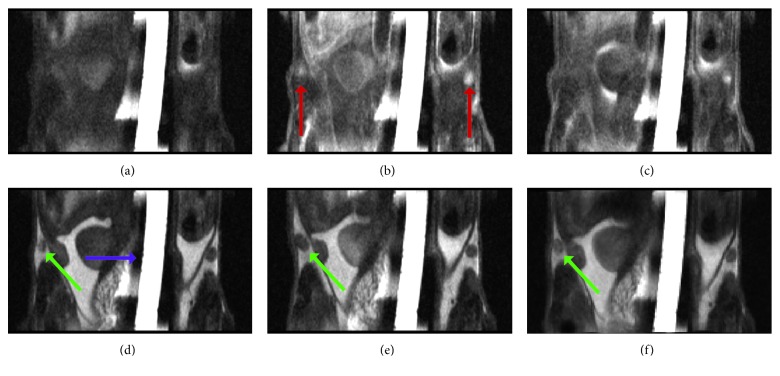
Precontrast (a), early (b), and late (c) enhancement of DCE-MRI images acquired, respectively, before 80 and 800 seconds after the administration of Gd-BOPTA and after a motion correction procedure. The lymph nodes are indicated by red arrows. Unsaturated fat T1-weighted images (same slice as (a)–(c)) before (d) and about 50 minutes after (e) the administration of the contrast agent, i.e., after the acquisition of the DCE-MRI scan; (f) is the same as (e) after a nonlinear motion correction procedure. Green arrows indicate a large displacement of the left node from (d) to (e) that was not fully recovered in (f) and consequently was excluded from the successive analysis. Frames (d)–(f) also show that a fatty hilum is not present. The white strip indicated by the violet arrow is a vial filled by gadolinium solution used as a standard signal in all DCE-MRI sequences.

Only superficial inguinal lymph nodes, clearly visible on DCE-MRI, were considered. Due to motion induced by breathing and bowel peristalsis during the MRI acquisition, the registration procedure was applied in 9 nodes. In 2 cases, the linear registration approach was not sufficient, being the images affected by bowel motion really close to the node, and they required nonlinear correction. In one case, motion produced a very large shift that was not fully recovered by the motion correction procedure, and the lymph node was excluded from the successive analysis. In total, 15 nodes were successfully identified and included in the analysis (9 by Gd-DTPA and 6 by Gd-BOPTA). Representative fat-suppressed DCE-MRI images of a healthy node are reported in Figures 1(a)–1(c), with the corresponding standard T1-weighted images (Figures 1(d)–1(f)). The node indicated by the green arrow was the one excluded from the analysis because of a clear displacement of its position during the scan session. Of note, within our spatial resolution, none of the observed lymph nodes showed a fatty hilum, as exemplarily shown in Figures 1(d)–1(f).

**Figure 2:**
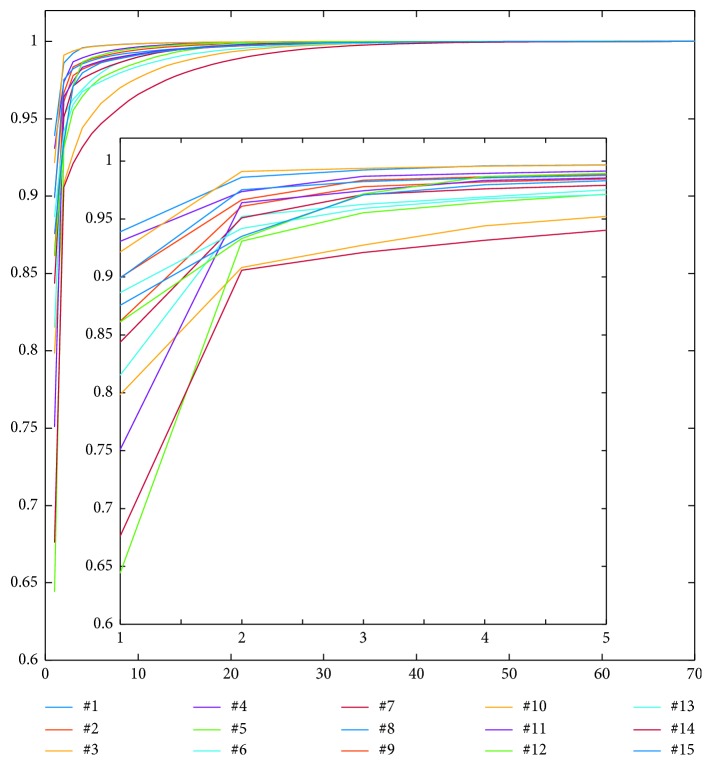
Principal component analysis (PCA) demonstrates that two principal components are sufficient to explain at least 90% of the variance in all the 15 nodes. Additional components negligibly increase variance explanation. The percentage of variance is reported in ordinate, and the component number (equal to the number of dynamic scans) is reported in abscissa. The whole range and a detailed range (in the inset) are shown.

**Figure 3:**
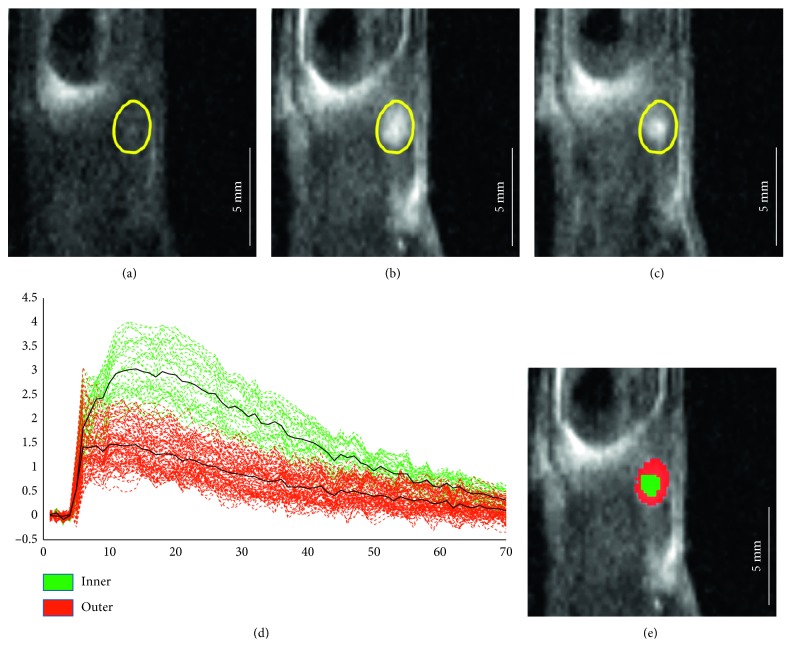
Precontrast (a), early (b), and late (c) DCE-MRI images (the same slice reported in Figures 1(a)–1(c) is shown) of a representative lymph node (#15). The region of interest (in yellow in (a)–(c)) was manually drawn to encompass the whole lymph node. The long-axis size of this lymph node was around 2.5 mm. k-means clustering assigned each pixel-based NDE curve (thin dashed lines) to one of the two clusters (d); cluster-averaged NDE curves are also shown (thick black continuous lines). The obtained segmentation map is shown with the corresponding colours (e).

Cluster analysis revealed a heterogeneous structure in the enhancement pattern of the lymph nodes. PCA performed on the NDE curves demonstrated that two main components were necessary and sufficient to explain at least 90% of the variance in all the 15 nodes (Figure 2). Additional components negligibly increased variance explanation. Consequently, the successive k-means clustering was performed by using two clusters. Of relevance, these two clusters were localized in the outer portion and in the inner portion of each lymph node in most of the investigated nodes. The spatial distribution and different enhancement of the identified clusters are shown in Figure 3, for a representative lymph node, with the corresponding description of the segmentation performed by k-means clustering. The curves clearly show different shapes, with the inner cluster being characterized by greater contrast uptake with respect to the outer cluster (Figure 3(d)).

**Figure 4:**
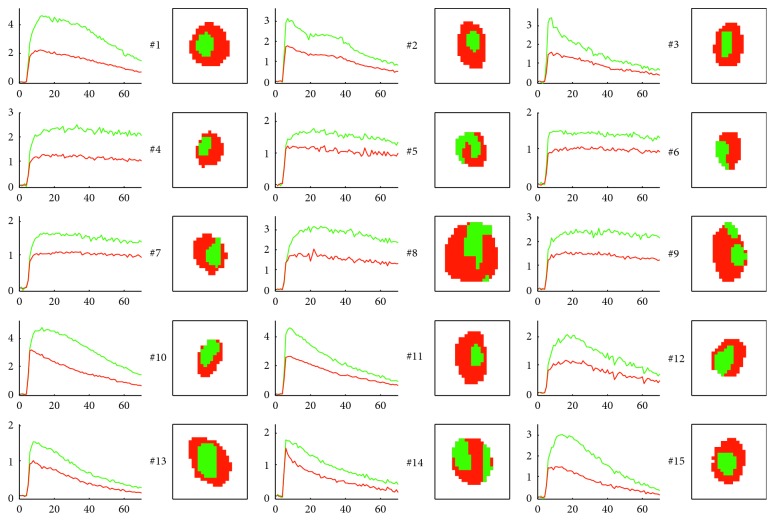
Enhancement pattern-based k-means clustering on 15 healthy nodes with Gd-DTPA (nodes #1–9) and with Gd-BOPTA (nodes #10–15). The red/green colours used to report the cluster-averaged NDE curves correspond to the colour used to localize them in the corresponding segmentation maps (on the right of each plot).

The cluster-averaged NDE curves and the corresponding coloured segmented maps of all the 15 examined lymph nodes are shown in Figure 4, confirming the peculiar heterogeneous enhancement pattern.

**Figure 5:**
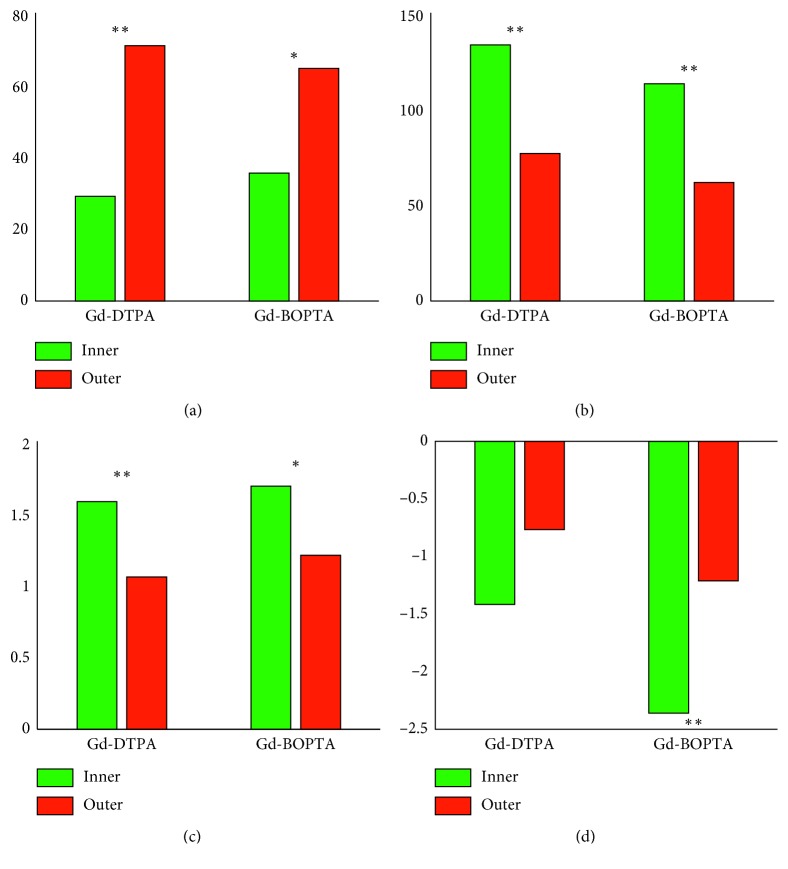
Mean parameter values calculated averaging on the lymph nodes evaluated with the same contrast agent. Statistical significance levels obtained comparing by two-way ANOVA the two segmented clusters are also shown (^*∗∗*^*p* < 0.01 and ^*∗*^*p* < 0.05). (a) Volume (%). (b) AUC (a.u.). (c) Wash-in rate (a.u./min). (d) Wash-out rate (a.u./min).

The parameters calculated on the cluster-averaged NDE curves are reported in Table 1, and the corresponding mean values are shown in Figure 5.

Two-way ANOVA was applied to compare the semiquantitative perfusion parameters averaged over the two clusters (significant differences are reported in Figure 5). As apparent from Figure 5, AUCs and wash-in rates were significantly smaller in the outer cluster, regardless of the administered contrast agent. The size of the outer cluster was significantly greater than the size of the inner cluster with either Gd-DTPA or Gd-BOPTA. The wash-out rates were significantly different between the two clusters with Gd-BOPTA, but close to be significant (*p*=0.056) with Gd-DTPA.

Two-way ANOVA performed through contrast agents revealed no statistically significant differences in the enhancement pattern of the two contrast agents tested here.

**Figure 6:**
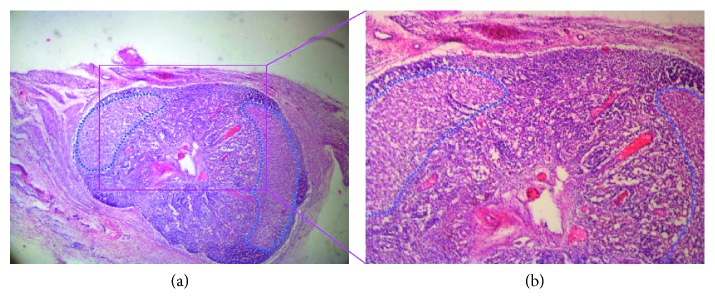
H&E staining of normal lymph nodes. (a) The lymphatic cell is clearly visible, and the lymph node structure is preserved both in the cortex (violet-surrounded area) and in the medulla (4x magnification). (b) 10x magnification of the boxed area shown in (a).

Histological analysis performed with H&E staining confirmed a heterogeneous structure of the normal node (Figures 6(a) and 6(b) (at 4x and 10x magnification)). The cortex (highlighted with blue dotted line in Figure 6) and the medulla of the node are clearly distinguishable.

## 4. Discussion

In this study, cluster analysis was applied on DCE-MRI data acquired on normal nodes of healthy mice by two Gd-based contrast agents characterized by different albumin-binding properties. k-Means clustering was applied to identify potential heterogeneities, and the minimum number of clusters was utilized in the algorithm. Of note, PCA suggested that most of the variability of the lymph node contrast enhancement could be accounted for by two main components with both contrast agents. The applied clustering method was based only on the enhancement pattern and not on the spatial position of each pixel assigned to the cluster; that is, neighborhood connectivity was not considered by the algorithm.

Of relevance, such a heterogeneous enhancement pattern arising from k-means evidenced a marked anatomical structure, regardless of the contrast agent used. In fact, the two clusters identified in each node resulted clearly localized in the outer portion and in the inner portion of the nodes, respectively. The observed pattern seems to be compatible with the functional organization of a normal lymph node, composed of two main functional units, the cortex and the medulla. Since the lymphatic flow is directed from the capsule and the cortex of the lymph node towards the medulla and the hilum, in this latter portion the contrast agents might accumulate also because of interstitial diffusion, at least partially explaining the greater contrast uptake in the inner region of the lymph nodes.

The observed spatial pattern characterized the heterogeneous distribution of the enhancement in all the assessed lymph nodes. Independently from the contrast agent used, AUC and wash-in rate were greater in the inner cluster, which also showed a steeper wash-out rate than the outer cluster both with Gd-BOPTA and by Gd-DTPA. On the contrary, the enhancement of Gd-DTPA was not significantly different from the enhancement pattern of Gd-BOPTA.

Many studies investigated the role of postcontrast T1 imaging in assessing the lymph node status, including quantitative assessment on DCE-MRI, but to our knowledge, the peculiar heterogeneity of contrast enhancement in normal nodes has not been reported before. To the best of our knowledge, only one study focused on normal nodes, analysing axillary lymph nodes in breast cancer patients [28]. However, that study focused on the enhancement curve of a single region of interest placed inside the cortex of the healthy nodes without covering the hilum to assess potential heterogeneity. As shown in native precontrast T1-weighted images, in our study, none of the assessed mice nodes showed a fatty hilum, allowing to include the whole nodes in the analysis of the contrast enhancement heterogeneity and the subsequent segmentation procedure.

Interestingly, in a study aiming at classifying axillary lymph nodes in breast cancer patients [18], the best performing feature was a morphological feature described as the degree to which the enhancement structure extends in a radial pattern originating from the centre of the node lesion. In our study, which of course needs to be confirmed on human subjects, an intrinsic radial structure on normal nodes was revealed, and it is reasonable to speculate that such a structure might be altered in metastatic nodes.

It is worth noting that the identification of a normal enhancement pattern might have a role in the detection of possible alterations induced by the metastatic transformation. For example, the accuracy obtained by lymphotropic iron oxide nanoparticles for nodal staging [29, 30] was based on the negative enhancement observed in normal nodes, which was modified by the presence of a metastatic lesion in the nodes. Similarly, in gadofosveset-enhanced MRI, the aspect of the chemical shift artefact encircling the lymph nodes (“the chemical shift criterion”) on enhanced images was considered a sign of benign normal nodes [31, 32]. Particularly, the benign nodes fell into the category of “sharply delineated and intact chemical shift artefact,” whereas an irregular or optically “interrupted” chemical shift artefact or a pronounced enhancing rim encircling the entire node was considered as a malignant criterion.

## 5. Conclusions

In conclusion, the results obtained showed a heterogeneous pattern of enhancement in normal lymph nodes, independently of the contrast agent used. Semiautomatic cluster analysis of signal intensity vs. time data showed the existence of two clusters characterized by different signal intensity dynamics belonging to the inner and outer regions of lymph nodes.

This heterogeneous pattern might be peculiar of normal lymph nodes and, if confirmed on human lymph nodes, should be deeply investigated to assess any alteration possibly induced by the metastatic transformation and, consequently, its potential role for nodal staging.

## Figures and Tables

**Table 1:** Semiquantitative parameters on segmented clusters.

	Node	AUC (a.u.)		Wash-in (a.u./min)		Wash-out (a.u./min)		Volume (%)	
		Inner	Outer	Inner	Outer	Inner	Outer	Inner	Outer
Gd-DTPA	#1	217 ± 41	100 ± 25	1.3 ± 0.3	0.8 ± 0.3	−3.8 ± 0.8	−1.5 ± 0.6	25	75
	#2	124 ± 16	71 ± 12	1.9 ± 0.3	1.2 ± 0.5	−3.0 ± 0.5	−1.6 ± 0.4	19	81
	#3	101 ± 14	58 ± 13	2.6 ± 0.4	1.2 ± 0.3	−1.9 ± 0.2	−1.2 ± 0.3	21	79
	#4	143 ± 23	74 ± 17	1.6 ± 0.5	0.9 ± 0.4	−0.6 ± 0.6	−0.5 ± 0.4	26	74
	#5	100 ± 9	69 ± 9	1.1 ± 0.7	1.1 ± 0.4	−0.7 ± 0.3	−0.4 ± 0.3	49	51
	#6	91 ± 9	63 ± 6	1.7 ± 0.5	1.2 ± 0.5	−0.5 ± 0.3	−0.3 ± 0.2	32	68
	#7	98 ± 11	66 ± 9	1.4 ± 0.4	1.2 ± 0.5	−0.7 ± 0.4	−0.4 ± 0.2	33	67
	#8	178 ± 26	99 ± 22	1.1 ± 0.3	1.0 ± 0.3	−1.1 ± 0.6	−0.5 ± 0.4	29	71
	#9	149 ± 27	91 ± 18	1.5 ± 0.5	1.0 ± 0.5	−0.4 ± 0.7	−0.6 ± 0.3	25	75

Gd-BOPTA	#10	211 ± 33	109 ± 29	1.4 ± 0.3	1.2 ± 0.2	−2.7 ± 0.5	−1.2 ± 0.4	47	53
	#11	157 ± 23	97 ± 14	2.3 ± 0.4	1.3 ± 0.4	−2.2 ± 0.6	−1.2 ± 0.3	14	86
	#12	88 ± 14	51 ± 12	1.3 ± 0.3	1.0 ± 0.3	−2.3 ± 1.2	−1.2 ± 0.7	40	60
	#13	51 ± 9	29 ± 7	1.9 ± 0.4	1.3 ± 0.4	−2.1 ± 0.4	−1.3 ± 0.3	38	62
	#14	61 ± 7	36 ± 8	1.5 ± 0.5	1.3 ± 0.7	−1.4 ± 0.2	−0.9 ± 0.3	43	57
	#15	109 ± 18	48 ± 16	1.7 ± 0.3	1.1 ± 0.4	−3.5 ± 0.8	−1.3 ± 0.5	29	71

## Data Availability

The MRI data used to support the findings of this study are available from the corresponding author upon request.
